# Alterations in regional homogeneity and multiple frequency amplitudes of low-frequency fluctuation in patients with new daily persistent headache: a resting-state functional magnetic resonance imaging study

**DOI:** 10.1186/s10194-023-01543-y

**Published:** 2023-02-23

**Authors:** Xueyan Zhang, Wei Wang, Xiaoyan Bai, Yanliang Mei, Hefei Tang, Ziyu Yuan, Xue Zhang, Zhiye Li, Peng Zhang, Zhangxuan Hu, Yaqing Zhang, Xueying Yu, Binbin Sui, Yonggang Wang

**Affiliations:** 1 Department of Neurology, The First Affiliated Hospital of Zhengzhou University, No.1 Jianshe East Road, Zhengzhou, China; 2 Headache Center, Department of Neurology, Beijing Tiantan Hospital, Capital Medical University, Beijing, China; 3 Tiantan Neuroimaging Center of Excellence, China National Clinical Research Center for Neurological Diseases, Beijing, China; 4 Department of Radiology, Beijing Tiantan Hospital, Capital Medical University, Beijing Neurosurgical Institute, Beijing, China; 5 GE Healthcare, Beijing, China

**Keywords:** New daily persistent headache, Regional homogeneity, Amplitude of low-frequency fluctuation, Multiple frequencies, Functional connectivity

## Abstract

**Background:**

New daily persistent headache (NPDH) is a rare primary headache that is highly disabling. The pathophysiology of NDPH is still unclear, and we aimed to reveal the underlying mechanism of NDPH through functional magnetic resonance imaging (fMRI) analysis.

**Methods:**

In this cross-sectional study, thirty patients with NDPH and 30 healthy controls (HCs) were recruited. The blood oxygen level-dependent (BOLD) sequences of all participants were obtained using the GE 3.0 T system. We performed ReHo, ALFF (conventional band: 0.01–0.08 Hz, slow-5: 0.01–0.027 Hz, slow-4: 0.027–0.073 Hz) and seed-based to the whole brain functional connectivity (FC) analysis in the NDPH and HC groups. The sex difference analysis of ReHo, ALFF, and FC values was conducted in the NDPH group. We also conducted Pearson’s correlation analysis between ReHo, ALFF, FC values and clinical characteristics (pain intensity, disease duration, HIT-6, GAD-7, PHQ-9, and PSQI scores).

**Results:**

Both increased ReHo (P_FWE-corr_ = 0.012) and ALFF values (0.01–0.08 Hz, P_FWE-corr_ = 0.009; 0.027–0.073 Hz, P_FWE-corr_ =0.044) of the left middle occipital gyrus (MOG_L) were found in the NDPH group compared to the HC group. There was no significant difference in FC maps between the two groups. Compared to the HC group, no difference was found in ReHo (*p* = 0.284), ALFF (*p* = 0.246), and FC (*p* = 0.118) z scores of the MOG_L in the NDPH group. There was also no sex difference in ReHo (*p* = 0.288), ALFF (*p* = 0.859), or FC z score (*p* = 0.118) of the MOG_L in patients with NDPH. There was no correlation between ReHo, ALFF, FC z scores and clinical characteristics after Bonferroni correction (*p* < 0.05/18).

**Conclusions:**

Patients with NDPH may have abnormal activation of the visual system. Abnormal visual activation may occur mainly in higher frequency band of the classical band. No sex differences in brain activity were found in patients with NDPH.

## Introduction

New daily persistent headache (NDPH) is a rare primary headache disorder with a prevalence of 0.03–0.1% in the general population [1]. NDPH was characterized by persistent headache and had clearly remembered onset, lasting over three months. There are no characteristic features of NDPH, and the headache may be migraine-like or tension-type-like [2]. NDPH is a highly disabling headache disorder due to unremitting pain, and psychiatric disorders are more prevalent in patients with NDPH than in healthy controls (HCs) [3–5].

The pathophysiology of NDPH is unclear. Structural and functional neuroimaging studies on NDPH are increasing but still insufficient. The resting-state fMRI approaches of regional homogeneity (ReHo) and amplitude of low-frequency fluctuation (ALFF) were used to depict the characteristics of brain functions [6, 7]. ReHo indicates regional homogeneity of spontaneous brain activities, and ALFF reflects the regional intensity of the blood oxygenation level-dependent (BOLD) signal. Previous rs-fMRI studies have revealed alterations in ReHo and ALFF in migraine [8–11]. However, the rs-fMRI studies of ReHo and ALFF in NDPH are still lacking. The BOLD signal of 0.01–0.08 Hz was thought to reflect spontaneous brain activities. Therefore, most ALFF studies in headache had chosen the conventional low-frequency band of 0.01–0.08 Hz [8, 9]. However, studies have suggested that different frequencies of neural oscillations in the brain may be sensitive to activity in different regions. Different frequencies of neural oscillations can be applied to indicate distinct physiological functions of brain activity [12, 13]. The frequency spectrum was divided into four different frequency bands, including slow-5 (0.01–0.027 Hz), slow-4 (0.027–0.073 Hz), slow-3 (0.073–0.198 Hz), and slow-2 (0.198–0.25 Hz). Slow-3 and slow-2 oscillations mainly reflect high-frequency physiological noise, low-frequency drift, and white matter signals. Slow-5 and slow-4 oscillations primarily reflect grey matter signals [13].

Compared to males, females were susceptible to pain perception and environmental stressors. The sex hormone levels were also different between males and females [14–16]. Significant sex differences have been found in several epidemiological studies of migraine [17–19]. NPDH has a female predominance, and the female-to-male gender ratio is 2.5:1 [20]. Enhanced recognition and implementation of attention to sex differences in human and animal research would help to strengthen and further our understanding of headache [21].

In this study, we calculated ReHo (0.01–0.08 Hz) and ALFF (0.01–0.08 Hz, 0.01–0.027 Hz, and 0.027–0.073 Hz) in the participants. Then, we chose the altered brain region identified by ReHo and ALFF analysis as the seed to process seed-based functional connectivity (FC) analysis. Additionally, we detected whether ReHo, ALFF, and FC values have sex differences in patients with NDPH. We also performed correlation analysis between the clinical characteristics of patients with NDPH and ReHo, ALFF, and FC values. We hypothesized that the ReHo, ALFF, and FC values were different in patients with NDPH compared to HCs, and the ReHo, ALFF, and FC values had sex differences in patients with NDPH. The ReHo, ALFF, and FC values in patients with NDPH correlated with the clinical characteristics.

## Methods

### Participants

In this cross-sectional study, patients diagnosed with NDPH at the Headache Center, Department of Neurology, Beijing Tiantan Hospital, Capital Medical University were enrolled. The diagnosis for each patient was confirmed by two senior neurologists according to the International Classification of Headache Disorders,3^rd^ version (ICHD-3). Thirty age- and gender-matched HCs were enrolled through recruitment advertisements and posters. All patients with NDPH had no medication overuse and were not taking any headache preventive medications. None of patients experienced NDPH after COVID-19 infection. General exclusion criteria for patients with NDPH and HCs were (1) combined with other headache, (2) combined with neurological or systematic disorders, (3) any drug or alcohol abuse history, and (4) not meeting the MRI safety screening criteria. The symptoms of anxiety and depression in HCs were assessed by psychiatrists from the multidisciplinary team (MDT) at Tiantan Hospital. HCs with anxiety and depression syndromes were excluded. We finally recruited 30 patients with NDPH and 30 HCs. The study was registered at https://www.clinicaltrials.gov (unique identifier: NCT05334927). The study was approved by the ethics committee of Beijing Tiantan Hospital, Capital Medical University (No. KY2022-044). All participants provided written informed consent.

### Questionnaires and scales

The Headache Impact Test (HIT-6) [22] score was used to determine the impact intensity of headache. Generalized Anxiety Disorder-7 (GAD-7) [23] and Patient Health Questionnaire-9 (PHQ-9) [24] scores were used to evaluate anxiety and depression symptoms. The Pittsburgh Sleep Quality Index (PSQI) [25] score was used to assess sleep quality.

### MRI acquisition

Images were obtained on a 3.0 T magnetic resonance scanner (Signa Premier, GE Healthcare; Chicago, Illinois, USA) through a 48-channel head coil at the National Neurological Center in Beijing Tiantan Hospital. Foam padding and earplugs were used to limit head movement and reduce the effect of noise on participants. All participants were instructed to hold a supine position, keep their eyes closed, and remain awake during the scan. The BOLD acquisition time was 330 s obtained using a multiband BOLD sequence with a 2.4 × 2.4 × 2.4 mm^3^ voxel size. The parameters were as follows: time point = 330, slice number = 65, flip angle = 64°, repetition time (TR) = 2000 ms, echo time (TE) = 68 ms, and field of view (FOV) = 208 mm × 208 mm. A high-resolution 3D T1-weighted structural image was acquired using the MP-RAGE sequence with 1.0 mm^3^ isotropic voxels. Its parameters were slice number = 192, flip angle = 8°, preparation time = 880 ms, recovery time = 400 ms, acquisition time = 240 ms, and FOV = 250 mm × 250 mm.

### Data processing

All imaging data were preprocessed and analysed using the toolkits of DPARSF (http://www.rfmri.org/DPARSF) and Statistical Parametric Mapping (SPM12) (http://www.fil.ion.ucl.ac.uk/spm) on a MATLAB (Mathworks, Natick, MA, USA) platform. Preprocessing procedures were as follows: (1) removal of the first ten volumes from 330 volumes; (2) slice timing correction; (3) realignment (the excessive head motion was defined as translation or rotation of > 2 mm or 2°); (4) coregistration; (5) nuisance covariates regression including linear trend, cerebral spinal fluid signals, white matter signals, and Friston-24 parameters of head motions; (6) spatial normalization into the Montreal Neurological Institute (MNI) space by DARTEL [26] with a resampled voxel size of 3 × 3 × 3 mm^3^; (7) data were filtered using the conventional band (0.01–0.08 Hz), slow-5 band (0.01–0.027 Hz), and slow-4 band (0.027–0.073 Hz); and (8) data smoothing with an 8-mm full width at half maximum (FWHM) Gaussian kernel.

### ReHo and multiple frequency ALFF calculations

The ReHo brain map was generated by calculating the concordance of Kendall’s coefficient of concordance (KCC) between each voxel and its 26 nearest neighbouring voxels using unsmoothed data (0.01–0.08 Hz). Then, to eliminate the effect of individual diversification, the KCC-ReHo value was normalized to the KCC-ReHo z value. A Gaussian kernel of 8 mm FWHM was used to spatially smooth the standardized ReHo maps [27]. After fast Fourier transformation, all voxels were converted from the time domain to the frequency domain. The averaged square root was obtained across three frequency bands (0.01–0.08 Hz, 0.01–0.027 Hz, 0.027–0.073 Hz) at each voxel as the ALFF value [28]. Then, all ALFF maps were converted into z-maps for standardization and prepared for subsequent statistical analysis.

### Seed-based whole-brain FC analysis

The brain regions for which ReHo and ALFF were commonly altered were selected as seed regions in the whole-brain FC analysis. The seeds were defined based on the automated anatomical labelling (AAL) atlas [29]. Then, we extracted the mean time course from the seed regions, and Pearson's correlation coefficients were calculated to correlate these time courses with whole-brain voxels. Finally, the FC maps were normalized into z score maps by Fisher Z-transformation.

### Statistical analysis

Statistical analyses were conducted using IBM SPSS software (version 21.0; Armonk, NY, USA). Descriptive statistics were summarized as the mean ± SD, and categorical variables were presented as frequency percentages. Independent sample t tests and chi-square analysis were used to compare continuous variables and categorical variables, respectively, between the NDPH and HC groups. We tested the data for normality through the Kruskal‒Wallis test before performing independent sample t tests. All statistical tests were two-tailed. The statistical significance was set at *p* < 0.05.

Statistical comparisons of ReHo, ALFF (0.01–0.08 Hz, 0.01–0.027 Hz, and 0.027–0.073 Hz), and FC values were conducted through a two-sample t test model generated in SPM12 software. The two-sample t test was performed at the voxel level with gender and age as covariates. The significance threshold was set at voxel-level *p* < 0.001 (two-tailed), and the familywise error (FWE)-corrected cluster extent threshold was* p* < 0.05. The z scores of mean ReHo, ALFF, and FC values of MOG_L in patients with NDPH and HCs were extracted. The z scores were used to compare group differences in mean ReHo, ALFF, and FC values between the NDPH and HC groups and gender differences in mean ReHo, ALFF, and FC values within the NDPH group through the independent sample t test. The significant difference was set at *p* < 0.05. Correlations between clinical characteristics and ReHo, ALFF, and FC values were calculated via Pearson’s correlation analysis. The significant difference was set at *p* < 0.05/18 (Bonferroni correction).

## Results

### Participant characteristics

The study included 30 patients with NDPH (15 males, 37.3 ± 10.8 years old) and 30 HCs (15 males, 38.3 ± 22.3 years old). No statistical power calculation was conducted before enrolling patients with NDPH. No participant was excluded after data quality control. There were no differences in age (*p* = 0.826), sex (*p* > 0.999), or BMI (*p* = 0.491) between the NDPH and HC groups. The VAS score was 5.0 ± 2.3, and the disease duration was 9.9 ± 12.4 years in patients with NDPH. Three patients with NDPH were unwilling to cooperate in completing the questionnaire and scale assessment, so HIT-6, GAD-7, PHQ-9, and PSQI scores were obtained from 27 patients with NDPH. Clinical characteristics for the NDPH and HC groups are displayed in Table 1.

**Table 1:** Demographic and clinical characteristics of healthy controls and patients with NDPH

	HCs (*n* = 30)	NDPH (*n* = 30)	*p*-value
Age (years)	37.3 ± 10.8	38.3 ± 22.3	0.826
Male patients, n (%)	15 (50.0)	15 (50.0)	> 0.999
BMI (kg/m^2^)	22.9 ± 3.1	23.5 ± 4.0	0.491
Pain intensity (VAS)	-	5.0 ± 2.3	-
Disease duration (years)	-	9.9 ± 12.4	-
Headache character, n (%)			
Throbbing	-	6 (20)	-
Pressing	-	7 (22.3)	-
Other	-	17 (55.7)	-
Associated headache symptoms, n (%)			
Nausea	-	8 (26.7)	-
Vomiting	-	3 (10.0)	-
Photophobia	-	15 (50.0)	-
Phonophobia	-	18 (60.0)	-
HIT-6 (36–78)^a^	-	62.9 ± 10.7	-
GAD-7 (0–21)^a^	-	6.8 ± 5.2	-
PHQ-9 (0–27)^a^	-	10.2 ± 7.0	-
PSQI (0–21)^a^	-	10.3 ± 5.1	-

*Abbreviation*: *NDPH* New daily persistent headache, *HCs* Healthy controls, *BMI* Body mass index, *VAS* Visual analogue scale, *HIT-6* Headache impact test, *GAD-7* Generalized anxiety disorder-7, *PHQ-9* Patient health questionnaire-9, *PSQI* Pittsburgh sleep quality index

^a^ Scales data were obtained from twenty-seven patients with NDPH

### ReHo and multiple frequency ALFF

**Fig. 1:**
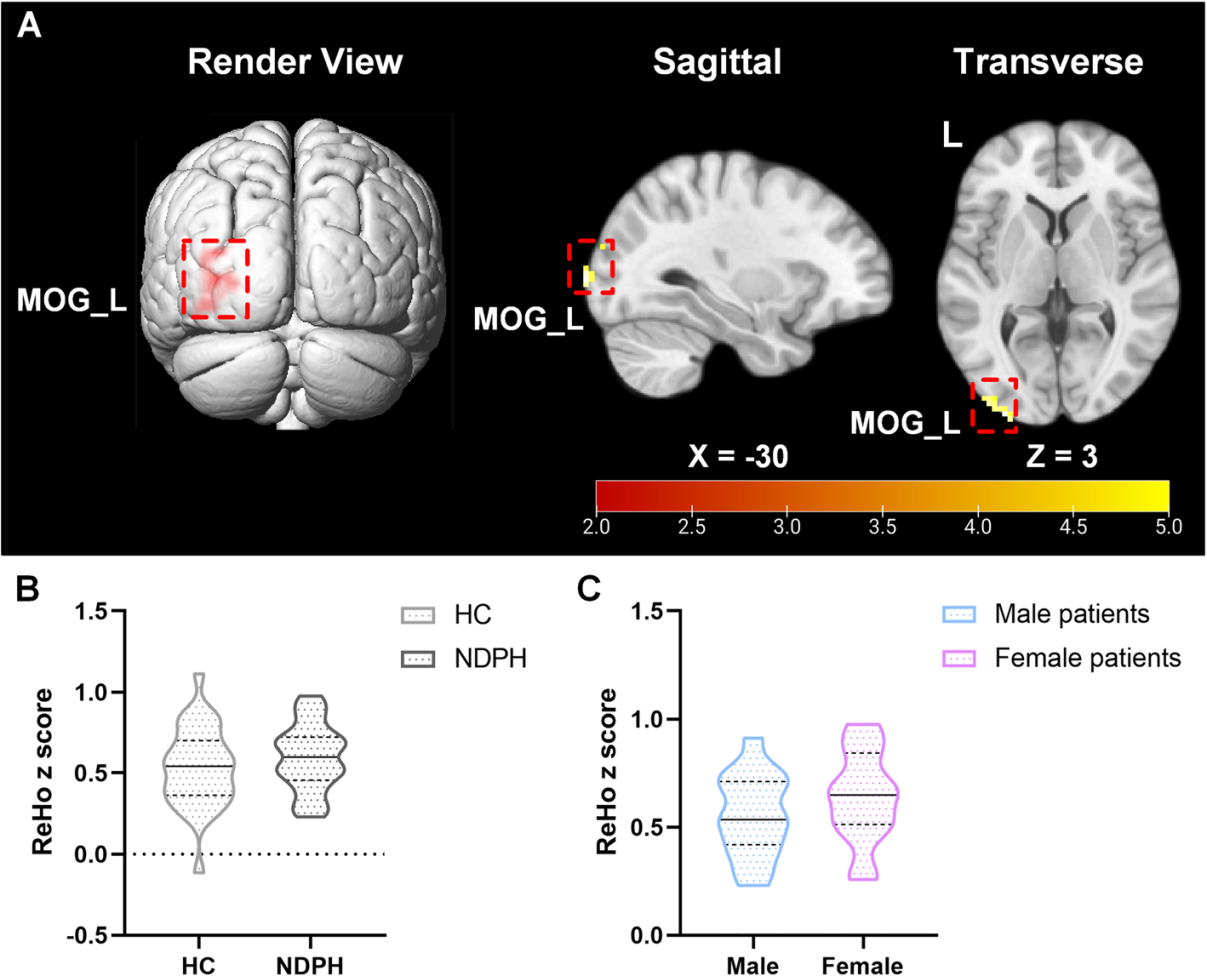
Results obtained from ReHo analysis (0.01–0.08 Hz) in the NDPH and HC groups. **A** The brain region showed significant group differences in ReHo analysis between the NDPH and HC groups. The colour bar represents the T values. **B** There was no significant difference in the ReHo z score between the NDPH and HC groups. **C** No sex difference in the ReHo z score was found in the NDPH group. Abbreviations: MOG, middle occipital gyrus; L, left; ReHo, regional homogeneity; NDPH, new daily persistent headache; HC, healthy control

The significant differences of ReHo values were presented in Table 2 and Fig. 1. Compared to the HC group, a two-sample t test of ReHo maps revealed that the NDPH group had increased ReHo values at the left middle occipital gyrus (MOG_L) (P_FWE-corr_ = 0.012). However, there was no difference in ReHo z score between the two groups (*p* = 0.284). There was also no sex difference of the ReHo z score in patients with NDPH (*p* = 0.288).

**Table 2:** Increased ReHo and ALFF values in the NDPH group compared to the HC group

Regions	MNI Coordinate			Cluster size	Peak Intensity	Cluster level P_FWE-corr_
	X	Y	Z			
**ReHo (0.01–0.08 Hz)**						
Cluster 1	-30	-99	3	69	4.49	0.012*
MOG_L						
**ALFF (0.01–0.08 Hz)**						
Cluster 1	-27	-102	3	45	5.36	0.009*
MOG_L						
**ALFF (0.027–0.073 Hz)**						
Cluster 1	-27	-102	3	32	5.16	0.044*
MOG_L						

*Abbreviations*: *ReHo* Regional homogeneity, *ALFF* Amplitude of low-frequency fluctuation, *NDPH* New daily persistent headache, *HC* Healthy control, *MNI* Montreal Neurological Institute, *FWE* Family-wise error; corr, correction, *MOG* Middle occipital gyrus, *L* Left

^*^ P_FWE-corr_ < 0.05, significance threshold was set at *p* < 0.05 (FWE cluster-level corrected) with a 30-cluster extension threshold

**Fig. 2:**
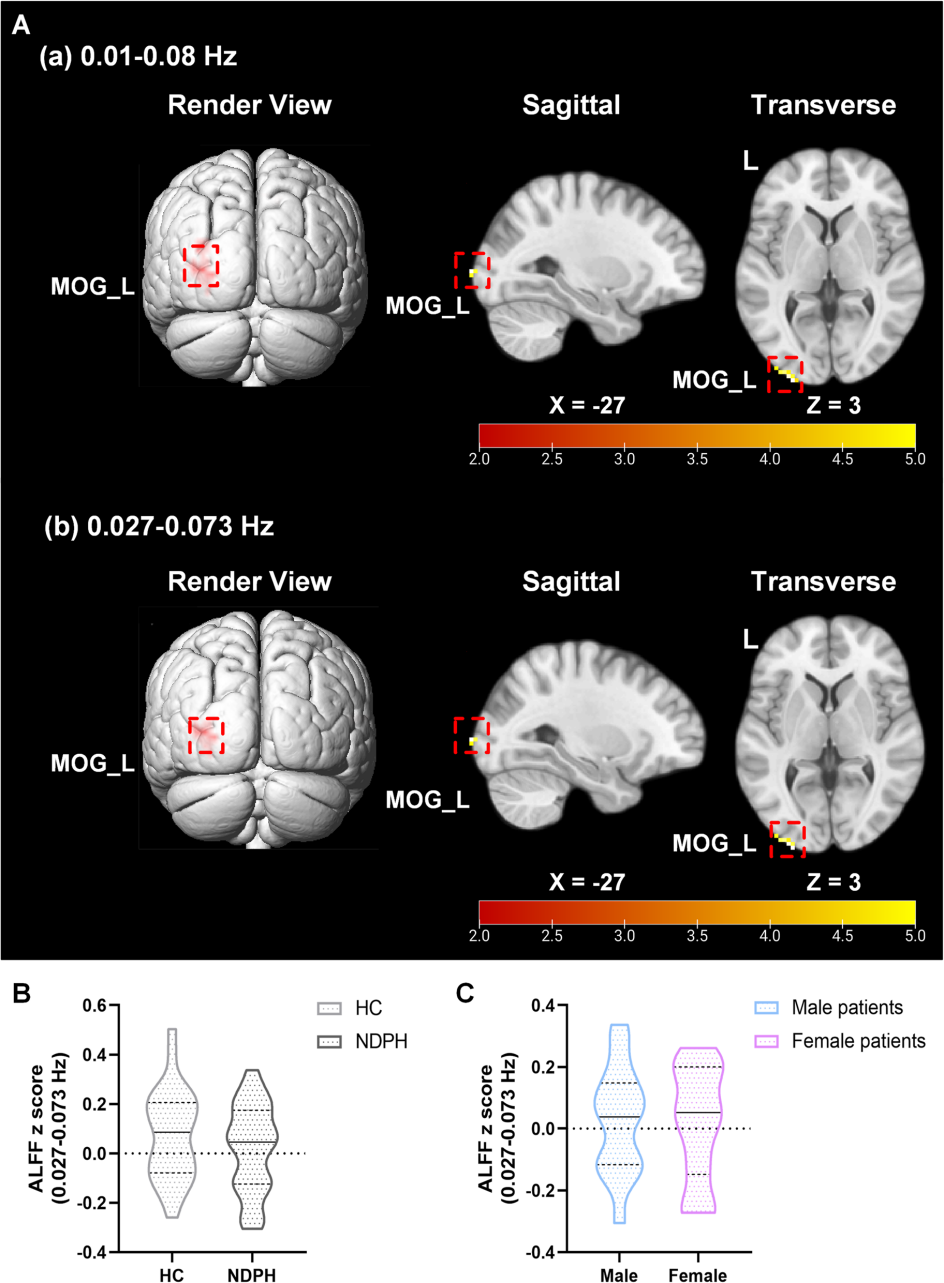
Results obtained from ALFF analysis (0.01–0.08 Hz, 0.027–0.073 Hz) in the NDPH and HC groups. **A** The brain region showed significant group differences in ALFF analysis in the 0.01–0.08 Hz (a) and 0.027–0.083 Hz (b) between the NDPH and HC groups. The colour bar represents the T values. **B** There was no significant difference in ALFF z scores in the 0.027–0.073 Hz between the NDPH and HC groups. **C** No sex difference in ALFF z score in 0.027–0.083 Hz was found in the NDPH group. Abbreviations: MOG, middle occipital gyrus; L, left; ALFF, amplitude of low-frequency fluctuation; NDPH, new daily persistent headache; HC, healthy control

The significant differences of multifrequency ALFF values are presented in Table 2 and Fig. 2. The two-sample t test of ALFF maps revealed that the NDPH group had increased ALFF values in the conventional band (0.01–0.08 Hz) (*p* = 0.009) and slow-4 band (0.027–0.073 Hz) (*p* = 0.044) at MOG_L compared to the HC group. In addition, there was no difference of ALFF values in the slow-5 band (0.01–0.027 Hz) between the two groups. There was no difference in the ALFF z score of the slow-4 band between the two groups (*p* = 0.246). There was also no sex difference of the ALFF z score (slow-4 band) in patients with NDPH (*p* = 0.859).

### Seed-based whole-brain FC

**Fig. 3:**
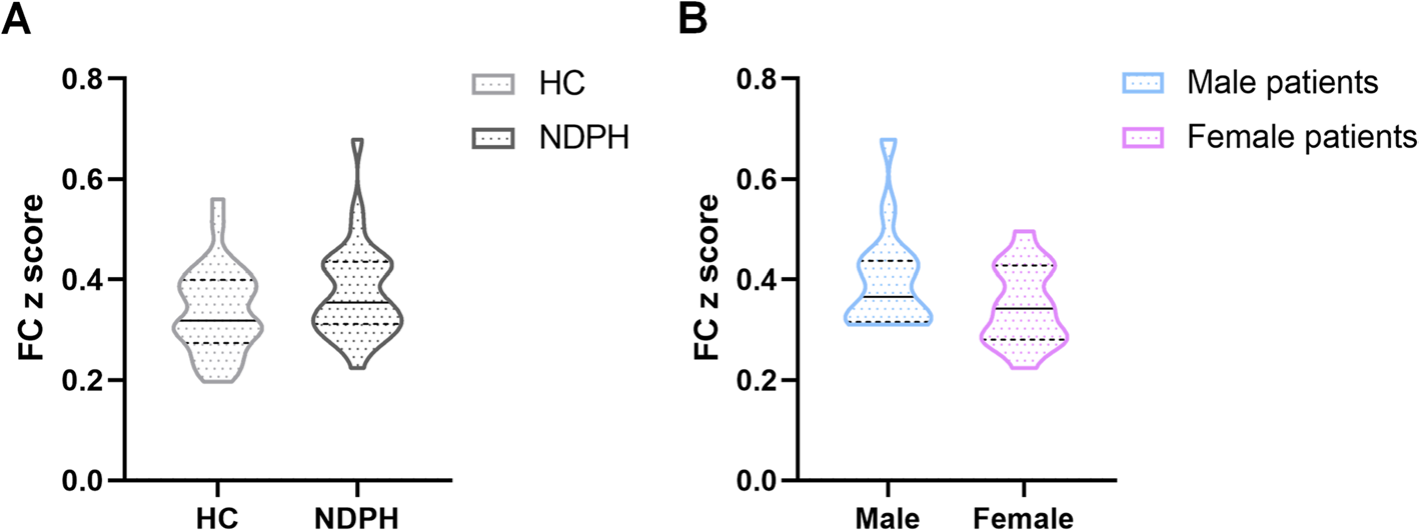
FC analysis from MOG_L to the whole brain voxels in the NDPH and HC groups. **A** There was no significant difference in the FC z score between the NDPH and HC groups. **B** No sex difference in the FC z score was found in the NDPH group. Abbreviations: FC, functional connectivity; MOG, middle occipital gyrus; L, left; NDPH, new daily persistent headache; HC, healthy control

We selected MOG_L based on the AAL atlas as the seed and progressed the seed to the whole voxel FC analysis, according to the ReHo and multifrequency ALFF results. There was no significant difference in FC maps between the NDPH and HC groups. There was no difference in the FC z score between the two groups (*p* = 0.134). There was also no sex difference in the FC z score in patients with NDPH (*p* = 0.118). The results are shown in Fig. 3.

### Correlation analysis

**Fig. 4:**
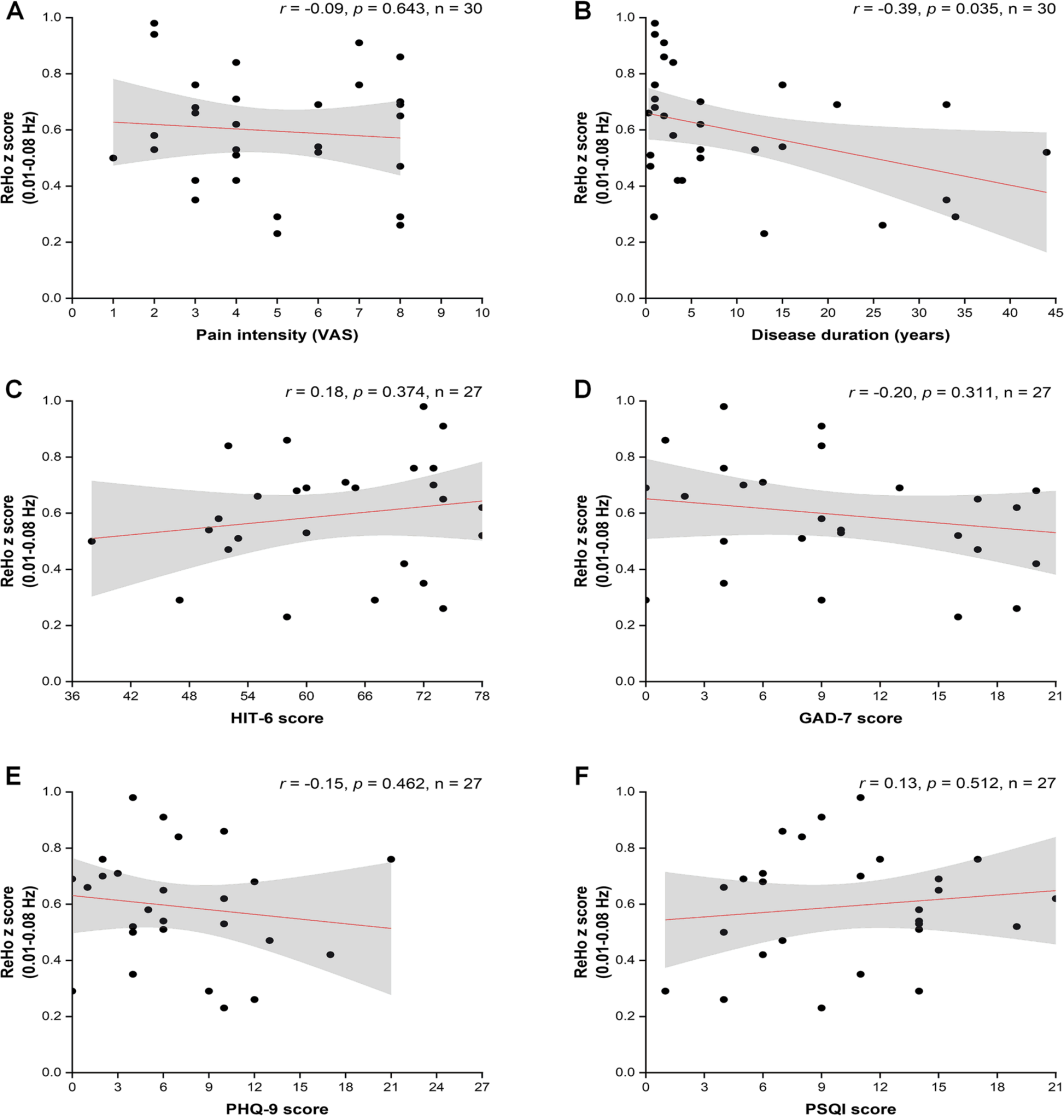
Correlation analysis between ReHo z score and clinical characteristics. **A**-**F** Correlation analysis between ReHo (0.01–0.08 Hz) z score and pain intensity, disease duration, HIT-6 score, GAD-7 score, PHQ-9 score, and PSQI score. There was no significant difference after Bonferroni correction (*p* < 0.05/18). Dots represent patients with NDPH. The red regression line represents Pearson’s correlation coefficient (r). Grey shading represents the 95% confidence intervals of the partial correlations. Abbreviations: ReHo, regional homogeneity; NDPH, new daily persistent headache; HC, healthy control; VAS, visual analogue scale; HIT-6, headache impact test; GAD-7, generalized anxiety disorder-7; PHQ-9, patient health questionnaire-9; PSQI, Pittsburgh sleep quality index

**Fig. 5:**
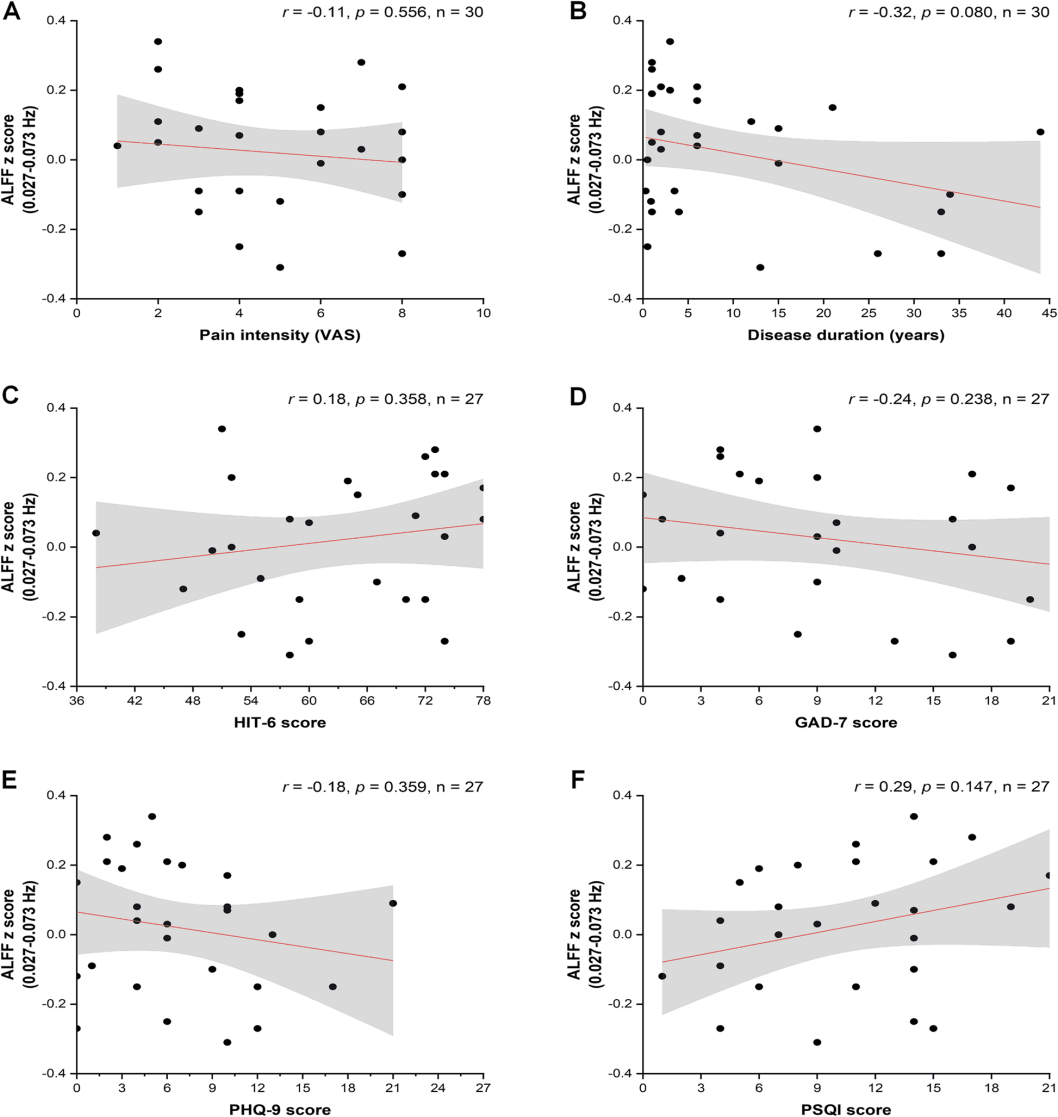
Correlation analysis between ALFF (0.027–0.073 Hz) z score and clinical characteristics. **A**-**F** Correlation analysis between ALFF (0.027–0.073 Hz) z score and pain intensity, disease duration, HIT-6 score, GAD-7 score, PHQ-9 score, and PSQI score. There was no significant difference after Bonferroni correction (*p* < 0.05/18). Dots represent patients with NDPH. The red regression line represents Pearson’s correlation coefficient (r). Grey shading represents the 95% confidence intervals of the partial correlations. Abbreviations: ReHo, regional homogeneity; NDPH, new daily persistent headache; HC, healthy control; VAS, visual analogue scale; HIT-6, headache impact test; GAD-7, generalized anxiety disorder-7; PHQ-9, patient health questionnaire-9; PSQI, Pittsburgh sleep quality index

**Fig. 6:**
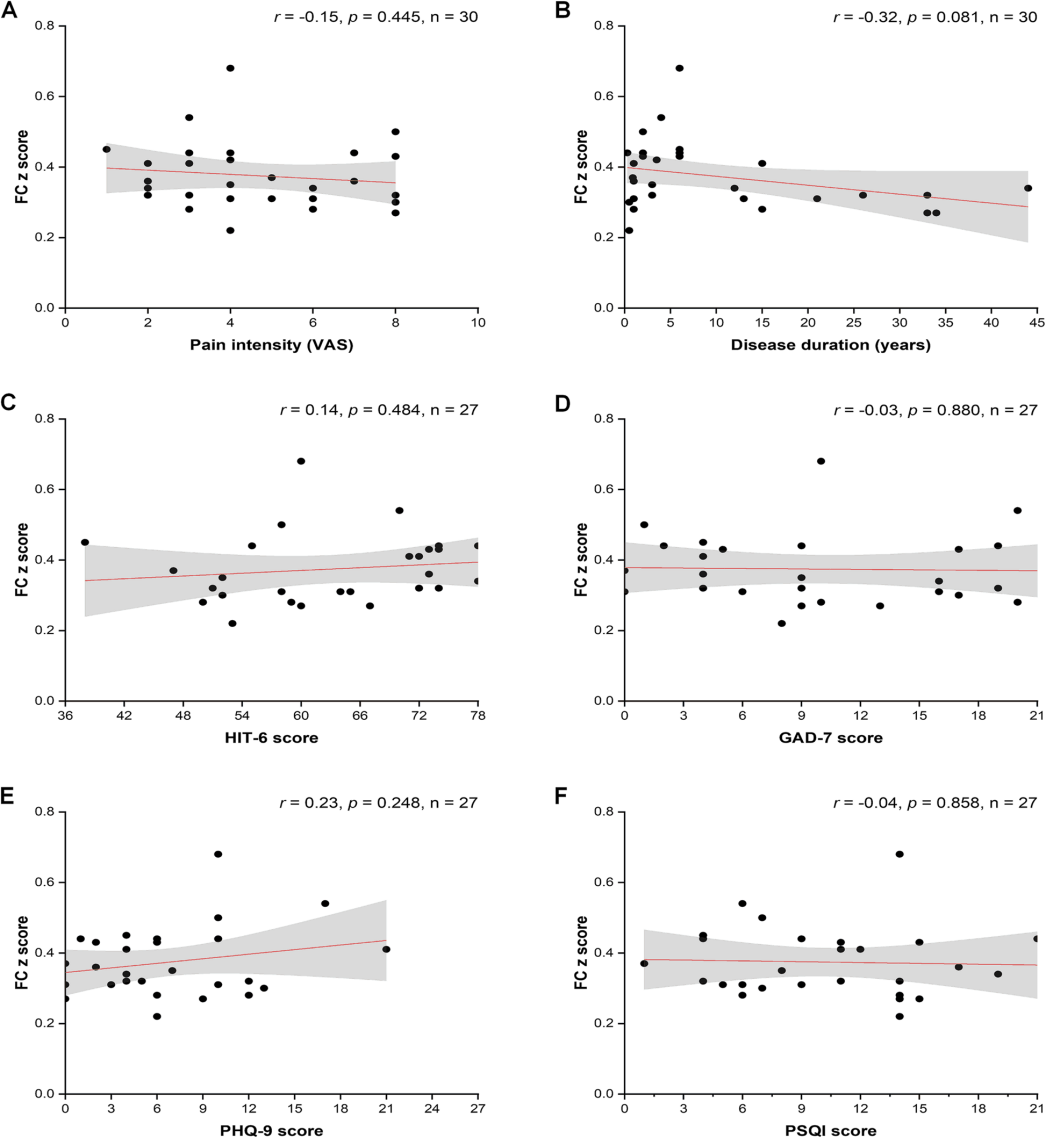
Correlation analysis between FC z score and clinical characteristics. **A**-**F** Correlation analysis between FC z score and pain intensity, disease duration, HIT-6 score, GAD-7 score, PHQ-9 score, and PSQI score. There was no significant difference after Bonferroni correction (*p* < 0.05/18). Dots represent patients with NDPH. The red regression line represents Pearson’s correlation coefficient (r). Grey shading represents the 95% confidence intervals of the partial correlations. Abbreviations: ReHo, regional homogeneity; NDPH, new daily persistent headache; HC, healthy control; VAS, visual analogue scale; HIT-6, headache impact test; GAD-7, generalized anxiety disorder-7; PHQ-9, patient health questionnaire-9; PSQI, Pittsburgh sleep quality index

The ReHo values were negatively correlated with disease duration (*r* = -0.39, *p* = 0.035). However, after Bonferroni correction (*p* < 0.05/18), there was no significant correlation between ReHo values (Fig. 4), ALFF values (slow-4) (Fig. 5), FC values (Fig. 6), and clinical characteristics (pain intensity, disease duration, HIT-6 score, GAD-7 score, PHQ-9 score, and PSQI score) (Table 3).

**Table 3:** Correlation between the ReHo, ALFF, and FC values of the left middle occipital gyrus and clinical characteristics

	ReHo (0.01–0.08 Hz)		ALFF (0.027–0.073 Hz)		FC	
	*r*	*p*	*r*	*p*	*r*	*p*
Pain intensity^b^	-0.09	0.643	-0.11	0.556	-0.15	0.445
Disease duration (years)^b^	-0.39	0.035*	-0.32	0.080	-0.32	0.081
HIT-6^a^	0.18	0.374	0.18	0.358	0.14	0.484
GAD-7^a^	-0.20	0.311	-0.24	0.238	-0.03	0.880
PHQ-9^a^	-0.15	0.462	-0.18	0.359	0.23	0.248
PSQI^a^	0.13	0.512	0.29	0.147	-0.04	0.858

*Abbreviations: ReHo* Regional homogeneity, *ALFF* Amplitude of low-frequency fluctuation, *FC* Functional connectivity, *HIT-6* Headache impact test, *PHQ-9* Patient health questionnaire-9, *GAD-7* Generalized anxiety disorder-7, *PSQ*I Pittsburgh sleep quality index

^*^ *p* < 0.05, there was no significant difference after Bonferroni correction (*p* < 0.05/18)

^a^ The scales data were obtained from twenty-seven patients with NDPH

^b^ The Headache characteristics data were obtained from thirty patients with NDPH

## Discussion

In our study, we found increased ReHo and ALFF (0.01–0.08 Hz, 0.027–0.073 Hz) values at MOG_L but no FC value difference via MOG_L to whole voxel FC analysis in the NDPH group compared to the HC group. We also found no sex difference through rs-fMRI analysis and no correlation with the clinical measures in patients with NDPH.

### Previous MRI studies in NDPH

A retrospective study on whether patients with NDPH develop white matter abnormalities or infarct-like lesions on neuroimaging involved 97 patients with NDPH found that only 13 patients with NDPH demonstrated white matter abnormalities, and none had infarct-like lesions [30]. Our previous arterial spin-labelled magnetic resonance imaging (MRI) study found decreased cerebral blood flow (CBF) and arterial cerebral blood volume (aCBV) values in the right posterior orbital gyrus, right middle occipital gyrus, and ventral anterior nucleus of the right thalamus in patients with NDPH (*n* = 15) compared to HCs (*n*= 15) [31]. A cross-sectional voxel-based morphometry (VBM) and surface-based morphometry (SBM) study investigated 23 patients with NDPH and 23 age- and gender-matched healthy controls (HCs) and found no grey matter changes in patients with NDPH [32]. Another cross-sectional study in 13 adolescents with NDPH found reduced cortical thickness in the bilateral superior temporal gyrus, left superior, and middle frontal gyrus areas and altered functional connectivity in the amygdala, insula, frontal regions, and cerebellar subregions compared to 13 HCs [33].

### Occipital and MOG in headache and pain

The occipital lobe is the centre of visual processing. It includes two functional units: the primary visual cortex (V1) and the visual associate cortex (V2, V3, V4) [34]. Previous magnetic resonance spectroscopy (MRS) studies found increased GLX [35, 36] and glutamate levels [37, 38], and decreased GABA levels [37, 39, 40] in the occipital lobe in patients with migraine. These studies revealed that alterations in the excitatory and inhibitory of the occipital lobe might play a significant role in migraine. Cortical spreading depression (CSD) is a slowly propagated wave of depolarization of neurons and glial cells, followed by a subsequent sustained suppression of spontaneous neuronal activity, accompanied by complex and variable changes in vascular caliber, blood flow, and energy metabolism [41]. Evidence from previous clinical and neuroimaging studies suggests that CSD is present in the occipital lobe of patients with migraine with aura [42]. Previous studies have shown MOG is the largest lobe of the occipital lobe, and alterations in MOG function have been identified in previous headache and pain neuroimaging studies. An fMRI study recruited seventy-two patients with migraine without aura and forty-six HCs and found decreased ReHo values of the left and right MOG in patients with migraine without aura [43]. Another study included 66 patients with chronic migraine (half with medication overuse) and 33 HCs and found decreased grey matter volume of MOG_L in patients with chronic migraine with medication overuse [44]. The study illustrated the previous findings that the occipital lobe participates in the aforementioned dopaminergic reward system [45, 46].

An ALFF study in patients with classical trigeminal neuralgia (CTN) included 48 patients with CTN found after a single trigger pain, and the ALFF values increased in the bilateral MOG in triggering-5 s and triggering-30 min [47]. A brain structure study via experimental induction of low back pain found that asymptomatic participants had greater grey matter density at MOG_L than participants with pain [48]. A study on alterations in local activity and functional connectivity in patients with postherpetic neuralgia after short-term spinal cord stimulation found patients with increased ReHo values at the MOG [49]. The visual system plays a prominent role in migraine, especially migraine with aura [50]. Previous studies have reported some patients with NDPH accompanied by photophobia symptoms [20]. In this study, 15 patients with NDPH had photophobia symptoms. However, based on our findings, it is difficult to explain the role of the visual system in NDPH patients. More clinical and basic experiments are needed to reveal the role played by the visual system in patients with NPDH.

### Slow-4 band ALFF

According to a previous MRI study, Slow-5 (0.01–0.027 Hz) and slow-4 (0.027–0.073 Hz) oscillations primarily reflect grey matter signals [13]. A study on Interictal brain activity differences in migraine with and without aura progressed through five frequency bands: 0.16–0.08 Hz, 0.08–0.04 Hz, 0.04–0.02 Hz, 0.02–0.01 Hz, and 0.01–0 Hz. The study found that all networks showed higher activity in the 0.08–0.04 Hz frequency range in patients with migraine with aura compared to patients with migraine without aura [51]. The study also found that the default mode network revealed decreased activity in the migraine without aura group in the 0.08–0.04 Hz band compared to the HC group. In our study, we divided the classical band (0.01–0.08 Hz) into slow-5 (0.01–0.027 Hz) and slow-4 (0.027–0.073 Hz). We also found the higher frequency slow-4 (0.027–0.073 Hz) had increased ALFF values at MOG_L in patients with NDPH. Higher frequency bands in the classical band may play a more important role in nociceptive regulation.

### No sex difference in brain activity in patients with NDPH

We found no sex difference in brain activity in patients with NDPH. On the one hand, this may be due to the lack of gender differences in brain activity in patients with NDPH, and on the other hand, it may be due to the limitations of our research methods. More new techniques may be needed to study this problem in depth in the future.

### Correlations with the clinical measures in patients with NDPH

NDPH is a highly disabling headache disorder due to unremitting pain. Psychological disorders are more prevalent in patients with NDPH than in HCs, such as anxiety and depression [4, 52]. Studies have reported that patients with NDPH have sleep disturbance [53, 54]. A previous study found no significant correlation between the functional connectivity results and Functional Disability Inventory and Pain Sensitivity Questionnaire scores of NDPH [33]. Our study found no significant correlation between fMRI results and any other clinical measures after Bonferroni correction. This may be associated with the participants having a long disease duration (9.9 ± 12.4 years) and being resilient to the impact of persistent pain and accompanying syndromes in NDPH.

### Limitations and future direction

We chose the altered brain region identified by ReHo and ALFF analysis to perform seed-based functional connectivity analysis. This approach may cause redundancy bias. The sample size was small, and the cluster size from the ReHo and ALFF analysis was also small, so the generalizability and specialty of the results may not be substantial. Patients with NDPH may have migraine-like or tension-type-like syndromes [55]. Investigating more homogeneous subgroups of patients with NDPH is needed. Additionally, the lack of questionnaires and scales for three patients with NDPH may have influenced the results of the correlation analysis. Future studies should explore FC analysis by an independent components analysis (ICA) approach or other fMRI paradigms assessing abnormal brain activation in patients with NDPH.

## Conclusions

Patients with NDPH may have abnormal activation of the visual system. Abnormal visual activation may occur mainly in higher frequency band of the classical band. No sex differences in brain activity were found in patients with NDPH.

## Data Availability

Data can be made available upon request.

## Abbreviations

NDPH: New daily persistent headache
VBM: Voxel-based morphometry
SBM: Surface-based morphometry
HCs: Healthy controls
MRI: Magnetic resonance imaging
CBF: Cerebral blood flow
aCBV: Arterial cerebral blood volume
ReHo: Regional homogeneity
ALFF: Amplitude of low-frequency fluctuation
BOLD: Blood oxygenation level dependent
FC: Functional connectivity
ICHD-3: The International Classification of Headache Disorders, 3^rd^ version
MDT: Multi-Disciplinary Team
TR: Repetition time
TE: Echo time
FOV: Field of view
SPM: Statistical parametric mapping
MNI: Montreal Neurological Institute
FWHM: Full width at half maximum
KCC: Kendall’s coefficient of concordance
AAL: Automated anatomical labeling
MOG_L: Left middle occipital gyrus
MRS: Magnetic resonance spectroscopy
CTN: Classical trigeminal neuralgia
CSD: Cortical spreading depression
ICA: Independent components analysis
